# 不分泌型多发性骨髓瘤的临床特征及预后分析

**DOI:** 10.3760/cma.j.issn.0253-2727.2022.10.011

**Published:** 2022-10

**Authors:** 子康 刘, 添丞 罗, 琬婷 强, 静 卢, 丽娜 金, 华 姜, 卫军 傅, 鹃 杜

**Affiliations:** 海军军医大学长征医院血液科、全军骨髓瘤与淋巴瘤疾病中心，上海 200003 Department of Hematology, the Myeloma & Lymphoma Center, Changzheng Hospital, Naval Medical University, Shanghai 200433, China

多发性骨髓瘤（MM）是一种单克隆浆细胞恶性增殖性疾病，以骨髓浆细胞异常增生伴单克隆免疫球蛋白生成为特征。国际骨髓瘤工作组将可检测的MM定义为血M蛋白≥1 g/dl和（或）尿M蛋白≥200 mg/24 h，如不满足上述标准，则需血清游离轻链（sFLC）≥100 mg/L（10 mg/dl）且sFLC比值异常[1]。血清和尿液免疫固定电泳M蛋白阴性，但骨髓穿刺浆细胞比例≥10％则称为不分泌型MM，血、尿M蛋白阳性但M蛋白量小于可测定范围称为寡分泌型MM。关于不分泌型MM患者的临床特征及预后与可检测的MM患者是否存在差异的研究目前报道尚少。近年来，蛋白酶体抑制剂（PIs）、免疫调节剂（IMiDs）等新药的出现使MM患者的总体预后得到极大改善[2]，本研究旨在评估新药时代我国不分泌型MM患者的临床特征及预后。

## 病例与方法

1. 病例：回顾性纳入2011年至2018年上海长征医院血液科新诊断的26例不分泌型MM患者，并通过医院系统随机抽取年龄匹配的260例非不分泌型MM患者（参照国内外诊断标准[3]–[4]）。鉴于不分泌型MM的定义，部分患者为M蛋白阳性但M蛋白不可测量的寡分泌型，本研究仅聚焦血清和尿免疫固定电泳呈阴性的患者[5]–[7]。

2. 间期FISH检测常见遗传学异常：首先抽取15～20 ml骨髓标本，肝素抗凝，采用密度梯度离心法分离骨髓单个核细胞，磁珠分离CD138阳性细胞，纯化后浆细胞低渗固定处理后收获细胞，−20 °C冻存。探针购自英国CYTOCELL公司，Ther-moBrite原位杂交仪购自美国自然基因有限公司。观察200个分裂间期细胞荧光杂交信号，进行图像分析（美国Video Test公司软件）。按照欧洲骨髓瘤工作组设定的阳性阈值标准，13q−、17p−和1q21扩增为阳性细胞百分率≥20％，融合基因为阳性细胞百分率≥10％。

3. 免疫固定电泳：采用美国Helena公司的Spife3000电泳仪及配套试剂检测患者外周血清，具体步骤包括琼脂糖凝胶电泳、免疫沉淀及显色三个步骤。阳性结果为在免疫固定电泳图谱上产生一条致密浓集的单克隆区带，并由专业技师判断结果检测报告，由此鉴定各种类型。

4. 治疗方案：本研究中，253例（88.5％）患者接受包含PIs的治疗方案［如硼替佐米+来那度胺+地塞米松（VRD）、硼替佐米+环磷酰胺+地塞米松（CBD）、硼替佐米+地塞米松（VD）、硼替佐米+沙利度胺+地塞米松（VTD）、硼替佐米+脂质体阿霉素+地塞米松（PAD）、伊沙佐米+来那度胺+地塞米松（IRD）等］，170例（59.4％）患者接受包含IMiDs的治疗方案［如VRD、VTD、CBD、沙利度胺+环磷酰胺+地塞米松（TCD）、来那度胺+地塞米松（RD）等］，57例（19.9％）患者接受了造血干细胞移植治疗。

5. 随访：随访截止日期为2021年8月，中位随访时间46.9（0.3～96.6）个月，以查阅患者住院病历及电话联系的方式进行随访。总生存（OS）期定义为自患者接受治疗到因任何原因死亡的时间，无进展生存（PFS）期定义为自患者接受治疗到疾病进展或因任何原因死亡的时间。

6. 统计学处理：采用SPSS 23.0和R version 4.1.1软件进行统计学分析。计数资料用例数（百分比）表示，计量资料用中位数（范围）表示。组间构成比、率的比较采用卡方检验，采用Kaplan-Meier法进行生存分析，Log-rank检验进行差异性分析。多因素分析采用Cox风险回归模型，*P*<0.05为差异有统计学意义。

## 结果

1. 临床特征：两组患者的临床特征见表1。≤65岁患者在不分泌型MM中所占比例低于非不分泌型MM患者（19.2％对32.7％，*P*＝0.159）。与非不分泌型MM患者相比，不分泌型MM患者女性比例（57.7％对35.8％，*P*＝0.047）、白蛋白中位水平（41.5 g/L对36.0 g/L，*P*<0.001）、血清LDH升高比例（36％对14.9％，*P*＝0.007）更高。不分泌型MM患者贫血（HGB≤100 g/L）（34.6％对61.9％，*P*＝0.007）、肾功能不全（DS分期B期，血肌酐≥177 µmol/L）（0％对23.1％，*P*＝0.006）、ISS Ⅲ期（15.4％对38.5％，*P*＝0.034）患者比例低于非不分泌型MM患者。

**表1:** 不分泌型与非不分泌型多发性骨髓瘤患者的临床特征比较

特征	总体（286例）	不分泌型（26例）	非不分泌型（260例）	统计量	*P*值
性别［例（％）］				3.946（*χ*^2^值）	0.047
男	178（62.2）	11（42.3）	167（64.2）		
女	108（37.8）	15（57.7）	93（35.8）		
年龄［岁，*M*（范围）］	60（23～83）	60（42～76）	60（23～83）	0.727（*t*值）	0.468
M蛋白类型［例（％）］					
IgA	61（21.3）				
IgD	27（9.4）				
IgG	121（42.3）				
不分泌型	26（9.1）				
轻链型	50（17.5）				
其他	1（0.4）				
DS分期［例（％）］				13.714（*χ*^2^值）	0.013
ⅠA	5（1.8）	0（0）	5（1.9）		1.000
ⅠB	1（0.4）	0（0）	1（0.4）		1.000
ⅡA	13（4.6）	0（0）	13（5.0）		0.616
ⅡB	2（0.7）	0（0）	2（0.8）		1.000
ⅢA	202（70.7）	26（100.0）	176（67.7）		<0.001
ⅢB	63（22.0）	0（0）	63（24.2）		0.002
ISS分期［例（％）］				5.548（*χ*^2^值）	0.062
Ⅰ	94（32.9）	12（46.2）	82（31.5）	2.288（*χ*^2^值）	0.130
Ⅱ	88（30.8）	10（38.5）	78（30.0）	0.794（*χ*^2^值）	0.373
Ⅲ	104（36.4）	4（15.4）	100（38.5）	4.488（χ^2^值）	0.034
R-ISS分期［例（％）］				2.880（*χ*^2^值）	0.237
Ⅰ	66（23.1）	8（30.8）	58（22.3）	0.953（*χ*^2^值）	0.329
Ⅱ	162（56.6）	14（53.8）	148（56.9）	0.091（*χ*^2^值）	0.763
Ⅲ	56（19.6）	2（7.7）	54（20.8）	2.567（*χ*^2^值）	0.109
数据缺失	2（0.7）	2（7.7）	0（0）	20.141（*χ*^2^值）	0.008
LDH［U/L，*M*（范围）］	162.5（66.0～819.0）	200.0（124.0～704.0）	160.0（66.0～819.0）	2017.5（*U*值）	0.004
骨髓浆细胞［％，*M*（范围）］	30.0（0.5～96.5）	30.0（1.5～93.5）	30.0（0.5～96.5）	3187.5（*U*值）	1.000
HGB［g/L，*M*（范围）］	93.0（38.0～156.0）	117.5（54.0～156.0）	89.0（38.0～151.0）	-4.026（*t*值）	<0.001
PLT［×10^9^/L，*M*（范围）］	169（13～413）	185（20～327）	164（13～413）	-1.114（*t*值）	0.266
血白蛋白［g/L，*M*（范围）］	37.0（14.0～79.0）	41.5（28.0～79.0）	36.0（14.0～54.0）	-4.537（*t*值）	<0.001
β_2_-微球蛋白［mg/L，*M*（范围）］	3.7（0.7～52.4）	2.8（0.8～8.5）	3.8（0.8～52.4）	2463.5（*U*值）	0.046
血钙［mmol/L，*M*（范围）］	2.4（1.8～3.8）	2.4（2.1～2.8）	2.4（1.8～3.8）	3088.0（*U*值）	0.468
血肌酐［µmoI/L，*M*（范围）］	77.0（5.2～1179.0）	66.5（32.0～88.0）	81.0（5.2～1179.0）	1820.0（*U*值）	<0.001
FISH［例（％）］					
17p−	35（12.3）	1（3.8）	34（9.2）	0.895（*χ*^2^值）	0.344
13q−	112（39.2）	8（30.8）	104（40.0）	0.409（*χ*^2^值）	0.523
1q21+	161（56.3）	13（50.0）	148（56.9）	0.068（*χ*^2^值）	0.794
t(11;14)	31（10.8）	6（23.1）	25（9.6）	5.878（*χ*^2^值）	0.015
t(4;14)	46（16.1）	1（3.8）	45（17.3）	1.742（*χ*^2^值）	0.187
t(14;16)	2（0.7）	1（3.8）	1（0.4）	0.768（*χ*^2^值）	0.381
数据缺失	2（0.7）	2（7.7）	0（0）		0.008
治疗方案［例（％）］					
包含蛋白酶体抑制剂	253（88.5）	25（96.2）	228（87.7）	0.933（*χ*^2^值）	0.334
包含免疫调节剂	170（59.4）	13（50.0）	157（60.4）	1.057（χ^2^值）	0.304
造血干细胞移植［例（％）］	57（19.9）	8（30.8）	49（18.9）	2.106（*χ*^2^值）	0.147

26例不分泌型MM患者中，4例sFLC数据缺失，22例患者中sFLC<100 mg/L患者19例（86.36％），其中sFLC比值正常患者10例（45.45％），为严格意义上的不分泌型MM；sFLC比值异常患者9例（40.91％），3例患者sFLC>100 mg/L且sFLC比值异常，受累游离轻链中位数2 355.2（587.6～7 269）mg/L，但患者血液和尿液免疫固定电泳均为阴性，本研究均归类为不分泌型MM。

不分泌型MM患者t（11;14）阳性比例明显高于非不分泌型MM患者（23.1％对9.6％，*P*＝0.015）。

2. 疗效：22例不分泌型MM患者进行了疗效评估，所有患者均接受包含PIs的治疗方案，13例（59.1％）达到完全缓解（CR），其中6例（46.2％）接受包含IMiDs的治疗；9例（40.9％）患者未达到CR，其中6例（66.7％）接受包含IMiDs的治疗。

3. 预后：本研究中位随访时间46.9个月，其中不分泌型MM患者的中位OS时间为87.8个月，中位PFS时间为37.8个月，1年、3年、5年OS率分别为88.1％、83.5％、74.2％；非不分泌型MM患者的中位OS时间为61.0个月，中位PFS时间为23.9个月，1年、3年、5年OS率分别为89.9％、67.2％、51.3％。单因素生存分析显示，两组患者OS（*P*＝0.155）及PFS（*P*＝0.370）的差异均无统计学意义（图1）。将年龄、性别、M蛋白类型、ISS分期、LDH水平、FISH检测结果等纳入多因素分析中，结果显示：影响OS的独立预后因素为ISS分期Ⅱ期、ISS分期Ⅲ期、LDH升高、17p−阳性、1q21+阳性；影响PFS的独立预后因素为ISS Ⅲ期、LDH升高、17p−阳性、1q21+阳性（表2）。但不分泌型并未展示出对PFS（*HR*＝0.701，95％ *CI* 0.329～1.496，*P*＝0.359）及OS（*HR*＝0.647，95％ *CI* 0.216～1.938，*P*＝0.437）的影响。

**图1:**
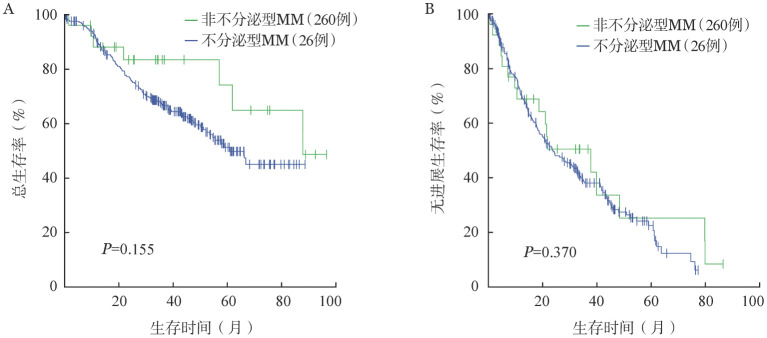
不分泌型与非不分泌型多发性骨髓瘤（MM）患者的总生存（A）与无进展生存（B）曲线

**表2:** 影响不分泌型多发性骨髓瘤患者总生存和无进展生存的多因素分析

因素	总生存		无进展生存	
	*HR*（95% *CI*）	*P*值	*HR*（95% *CI*）	*P*值
ISS分期（Ⅱ期，Ⅰ期）	1.902（1.052～3.441）	0.033		
ISS分期（Ⅲ期，Ⅰ期）	2.360（1.296～4.298）	0.005	1.782（1.151～2.761）	0.010
LDH（升高，正常）	1.002（1.001～1.004）	0.005	1.002（1.001～1.003）	0.003
17p−（阳性，阴性）	2.225（1.302～3.801）	0.003	1.662（1.045～2.644）	0.032
1q21+（阳性，阴性）	1.792（1.130～2.841）	0.013	1.882（1.302～2.720）	<0.001

## 讨论

本研究分析了26例不分泌型MM患者的临床特征及预后，并与非不分泌型MM进行比较，观察到不分泌型MM与非不分泌型MM的临床特征存在差异，但预后无明显差异。

不分泌型MM是MM中的一种少见亚型，国际骨髓瘤工作组将血、尿免疫固定电泳M蛋白阴性的MM定义为不分泌型MM[3]。随着新检验技术的出现，一部分既往被认为是不分泌型MM的患者可检测到sFLC异常，部分研究将其定义为寡分泌型MM[8]。目前对于如何更准确地定义不分泌型MM仍未统一。不分泌型MM为罕见类型，既往研究由于界定标准不同，发病率为1％～5％[5]–[7],[9]。血、尿中免疫固定电泳M蛋白呈阴性且sFLC无异常的严格意义的不分泌型MM有3种情况：①浆细胞不产生任何免疫球蛋白；②浆细胞产生免疫球蛋白但不能分泌；③浆细胞产生且分泌免疫球蛋白，仅根据病理证实免疫球蛋白沉积，但常规手段无法检测出[10]–[12]。不分泌型MM的发病机制尚未明确，可能的原因及假说包括：多聚腺苷酸化位点缺失、重链V区缺失、轻链突变等[12]–[14]。

本研究中不分泌型MM患者的临床特征表现为贫血、肾功能不全，DS分期ⅢB期患者比例较低，由此可见，不分泌型MM在诊断时肿瘤负荷低于非不分泌型MM。FISH结果显示，不分泌型MM患者t（11;14）阳性比例较高，与既往研究结果相同[7]。尤为重要的是，新型药物Bcl-2抑制剂（维奈克拉）对t（11;14）具有较好疗效[15]。

既往报道显示，不分泌型MM患者的预后差异较大[5]–[7]。本研究中，不分泌型MM患者的中位OS时间长于非不分泌型MM患者，但单因素及多因素预后分析均提示两组患者预后差异无统计学意义。既往研究中，传统化疗时代不分泌型MM患者的中位OS时间为43.2个月[5]，本研究中不分泌型MM患者的中位OS时间为87.8个月，由于本研究中不分泌型MM患者均接受了PIs、IMiDs等新型药物治疗，提示新药较传统药物能显著延长不分泌型MM患者的生存时间。不分泌型与非不分泌型MM的发病机制及生物学特征差异仍需深入研究。

总之，本研究观察到不分泌型MM患者在临床特点上表现为肿瘤负荷较小，新药时代不分泌型MM患者的生存时间较传统药物治疗时代显著延长，其预后与非不分泌型患者无显著差异，其发病机制仍需深入探索。
